# Albumin, mNUTRIC and NRS-2002: Predicting Mortality in Elderly ICU Fracture Patients

**DOI:** 10.3390/healthcare14111431

**Published:** 2026-05-22

**Authors:** Hatice Zeynep Atlı, Osman Yağız Atlı, Ayşe Müge Karcıoğlu, Merve Tokatlı Doğan, Gözde Şengül Ayçicek, Semih Aydemir, Mesher Ensarioğlu, Onur Küçük, Yavuz Kutay Gökçe

**Affiliations:** 1 Department of Intensive Care, Ankara Etlik State Hospital, 06170 Ankara, Türkiye; 2 Department of Orthopedy and Traumatology, Ankara Etlik State Hospital, 06170 Ankara, Türkiye; 3 İstanbul Provincial Health Directorate, 34122 İstanbul, Türkiye; 4 Department of Geriatric Medicine, Ankara Etlik State Hospital, 06170 Ankara, Türkiye; 5 Department of Anesthesiology and Reanimation, Ankara Yenimahalle Training and Research Hospital, University of Yıldırım Beyazit, 06370 Ankara, Türkiye; 6 Department of Anesthesiology and Reanimation, Ankara Gülhane Training and Research Hospital, 06010 Ankara, Türkiye; 7 Department of Anesthesiology and Reanimation, Faculty of Medicine, Dokuz Eylül University, 35340 İzmir, Türkiye; 8 Department of Internal Medicine, Ankara Yenimahalle Training and Research Hospital, 06200 Ankara, Türkiye

**Keywords:** serum albumin, malnutrition, nutritional risk, older adult fracture, intensive care unit, mortality

## Abstract

Objective: The primary objective was to evaluate whether admission serum albumin predicts six-month all-cause mortality in older adult patients admitted to the intensive care unit (ICU) after simple fracture surgery, and to compare its predictive performance with the modified Nutrition Risk in the Critically Ill (mNUTRIC) score and the Nutrition Risk Screening 2002 (NRS-2002). The secondary objectives were to identify baseline predictors of six-month mortality and high-risk mNUTRIC classification. Methods: This retrospective cohort study included patients aged ≥65 years admitted to the ICU of a tertiary care hospital after surgery for a simple fracture between July and December 2024. Demographic data, comorbidities, admission laboratory values (including albumin, prealbumin, and 25-hydroxy vitamin D, the latter included as an adjunctive nutritional biomarker), APACHE II, SOFA, mNUTRIC, and NRS-2002 scores were recorded. Postoperative complications and admission durations were evaluated. Binomial logistic regression models were constructed for six-month all-cause mortality and nutritional risk group classification. Receiver operating characteristic (ROC) analysis with the Youden Index was performed to determine cutoff values. Results: A total of 172 patients (mean age 80.84 ± 7.72 years; 67.4% female) were analyzed. Six-month all-cause mortality was 22.7%. Serum albumin (OR 0.823, 95% CI 0.729–0.928, *p* = 0.002) and ICU admission duration (OR 1.413, 95% CI 1.101–1.812, *p* = 0.007) were independent predictors of six-month all-cause mortality, whereas mNUTRIC, NRS-2002, and vitamin D were not. Neither mNUTRIC nor NRS-2002 scores differed significantly between survivors and non-survivors. In nutritional risk group analysis, age (OR 1.117, *p* = 0.001) and APACHE II (OR 1.694, *p* = 0.001) were independent predictors of high mNUTRIC risk. Head-to-head ROC analysis for the primary outcome of six-month all-cause mortality showed that admission serum albumin (AUC 0.698, 95% CI 0.604–0.793) provided significantly better discrimination than mNUTRIC (AUC 0.570, DeLong *p* = 0.046) and NRS-2002 (AUC 0.550, DeLong *p* = 0.039). In a sensitivity model restricted to admission-time variables (albumin, age, APACHE II, vitamin D, Charlson Comorbidity Index), admission albumin remained an independent predictor (OR 0.830, 95% CI 0.747–0.923, *p* < 0.001) and age emerged as a further independent predictor (OR 1.062, *p* = 0.034). Conclusions: Serum albumin outperformed mNUTRIC and NRS-2002 in predicting six-month all-cause mortality among older adult post-fracture ICU patients. Because neither mNUTRIC nor NRS-2002 discriminated between survivors and non-survivors, these scores alone cannot be recommended as mortality-prediction tools in this orthogeriatric ICU population. Whether admission albumin adds incremental value to existing nutritional scoring in this setting requires prospective, adequately powered validation.

## 1. Introduction

Malnutrition is a state defined by changes in body and tissue composition arising from deficiencies in protein, energy, or other nutrients [1]. Its possible clinical consequences are evident in the population, especially among those considered critically ill. Patients in the acute phase of illness or the surgical recovery period may be more affected by nutritional status due to stress-induced increases in metabolism and protein catabolism [2]. Clinical studies have presented malnutrition as a significant problem in overall patient care, with increased complications, including susceptibility to infection, delayed wound healing, and prolonged hospitalization, and have proposed interventions when required [3,4].

Numerous scoring systems have been proposed to identify malnutrition. These include the Nutrition Risk Screening 2002 (NRS-2002), the Malnutrition Universal Screening Tool (MUST), and the Nutrition Risk in the Critically Ill (NUTRIC) score [5]. NRS-2002 uses body weight and body mass index (BMI) as part of its assessment, which may be misleading in some critically ill patients who may require extensive fluid therapy [6]. The NUTRIC score is a commonly used tool for assessing malnutrition, developed by Heyland et al. in 2011, and incorporates age, Acute Physiology and Chronic Health Evaluation II (APACHE II), Sequential Organ Failure Assessment (SOFA), comorbidities, length of stay in the intensive care unit, and serum interleukin-6 (IL-6) levels [7]. Patients with a score of 6 or higher out of 10 in this scoring system are classified as high risk. However, the presence of IL-6 in the system may lead to practical difficulties. To address possible limitations of NUTRIC due to IL-6, Rahman et al. proposed a modified version of the NUTRIC score, known as modified NUTRIC (mNUTRIC), which excludes IL-6 from assessment [8]. This modified score has been reported to have comparable sensitivity to the original score, albeit at a different threshold of 5 for grouping patients as high risk. Although developed for different target populations (NRS-2002 originally as a general hospital admission screening tool endorsed by the European Society for Clinical Nutrition and Metabolism (ESPEN) [6], and mNUTRIC specifically for the intensive care setting [8]), both have been extensively applied and compared in adult ICU practice. These two scores were selected as comparators against admission serum albumin in the present study because together they represent the two most commonly applied nutritional risk instruments in this setting: one reflecting the ESPEN-endorsed hospital admission standard, the other purpose-built for the critically ill.

Ortopedic intervention among older adult patients is a topic of interest regarding possible modalities that could be used to reduce overall mortality and morbidity, especially regarding intensive care outcomes [9]. Studies that have utilized different laboratory markers and anthropometric measures are reported, with different methodologies being suggested depending on the planned surgery [10]. Meermans et al. studied the role of malnutrition on hip fracture patients, with Cacciola et al. investigating the role of laboratory values and their respective ratios upon nutritional prediction [11,12]. New mortality studies focusing on hip surgery outcomes that incorporate nutritional assessment are also proposed [13]. Further studies are required as traditional markers and assessment methods may fail for specific groups of patients [14]. Studies, however, are limited in the assessment of simple fractures, especially those of the lower extremities, regarding nutritional outcome among older adults. Among individual biomarkers, serum albumin has been extensively studied as a global indicator of nutritional and inflammatory status in geriatric fracture patients. 25-hydroxy vitamin D has also attracted attention in this population. Through vitamin D receptor signaling in skeletal muscle, it influences myocyte function and neuromuscular performance; deficiency in older adults has consequently been linked to sarcopenia and loss of muscle strength, to postural instability and a higher risk of falls, and to impaired postoperative musculoskeletal recovery—mechanisms of particular relevance in frail orthogeriatric patients, in whom 25-hydroxy vitamin D deficiency is highly prevalent and frequently severe [15]. Its potential role as an adjunctive prognostic marker in orthogeriatric ICU patients, however, remains insufficiently characterized, which motivated its inclusion in the present analysis.

The primary aim of this study was to determine whether admission serum albumin predicts six-month all-cause mortality in older adult patients admitted to the ICU after simple fracture surgery, and to compare its predictive performance with that of the mNUTRIC score and NRS-2002. As for secondary goals, the aim was to identify independent predictors of six-month all-cause mortality and classification into the high-risk mNUTRIC group, and to assess the performance of routinely collected scores (APACHE II, SOFA) in the orthogeriatric ICU cohort. It was hypothesized that admission serum albumin, a simple, widely available biomarker, would outperform both mNUTRIC and NRS-2002 in discriminating six-month all-cause mortality in this population, with predictive performance compared head-to-head using the area under the receiver operating characteristic (ROC) curve (AUC) and pairwise AUC comparison via DeLong’s test.

## 2. Materials and Methods

The study was conducted as a retrospective cohort study in the intensive care units of a tertiary care hospital. The study population included patients admitted postoperatively from July 2024 to December 2024. It was initiated after receiving ethics approval (Approval date: 26 March 2025, approval number: AEŞH-BADEK-2025-0479, Ethics Committee: Ankara State Hospital Scientific Research Evaluation and Ethics Committee) and was carried out in accordance with the Declaration of Helsinki. The inclusion criteria were age 65 years or older and admission to an intensive care unit following surgery for a simple fracture. Exclusion criteria included being under 65, having a multitrauma diagnosis, missing prealbumin and vitamin D results upon admission, or experiencing high-energy trauma. A simple fracture was defined as an isolated fracture of a single bone or anatomical region (e.g., proximal femur, humerus, tibia) resulting from low-energy trauma such as a fall from standing height. High-energy trauma was defined as injury resulting from a mechanism capable of transferring substantial kinetic energy to the skeleton, including motor vehicle collisions, pedestrian versus vehicle injuries, falls from a height greater than three meters, or direct crush injuries. Patients meeting any of these criteria were excluded, as the systemic inflammatory and metabolic response in such cases differs substantially from that of frailty-related low-energy fractures and would confound the nutritional assessment under investigation. Patients with fractures in two or more different anatomical regions, or with concomitant head, thoracic, or abdominal trauma, were classified as multitrauma and excluded.

Patients’ demographic characteristics, comorbidities, and Charlson Comorbidity Index score, fracture location, and admission laboratory values (including blood cell count, inflammatory markers, renal function tests, albumin, prealbumin, venous blood gas parameters (pH, pCO2, HCO3, lactate), electrolytes (sodium, potassium, calcium, magnesium), and vitamin D levels) were recorded. To minimize the risk of selection bias inherent in retrospective laboratory data, only the first set of values obtained within the initial 24 h of ICU admission was used for analysis; later measurements, peak values, or time-averaged values were not considered. When more than one result was available within this 24 h window (for example, repeat venous blood gases), the earliest available value was taken. 25-hydroxy vitamin D and prealbumin were measured once on ICU admission as part of the routine orthogeriatric admission panel at our institution and were not repeated. This single-time-point approach was applied uniformly to all patients. Mortality (APACHE II and Sequential Organ Failure Assessment-SOFA) and nutritional (Modified Nutrition Risk in the Critically Ill-NUTRIC and Nutrition Risk Screening 2002-NRS 2002) outcomes were also recorded. Intensive care unit parameters, including admission duration, presence of any complication, and additional support requirement and duration, were recorded, with total admission and ward admission duration also included in the evaluation. “Any complication” was defined as the occurrence of one or more of the following prespecified postoperative events during the index hospitalization: acute renal failure (defined by KDIGO criteria), clinically significant postoperative hemorrhage requiring transfusion or surgical revision, hypotension requiring vasopressor support, acute respiratory failure requiring supplemental oxygen beyond routine postoperative care or mechanical ventilation, and sepsis. Each event was also analyzed separately. The primary outcome was all-cause mortality at six months from the date of ICU admission (i.e., within 180 days of ICU admission), ascertained from the national death registry and confirmed through hospital records. Six-month mortality status was ascertained from the Turkish Ministry of Health Death Notification System (Ölüm Bildirim Sistemi, ÖBS), which is the operational interface to the Central Civil Registration System (MERNIS). Records were accessed through the hospital information system (HBYS) under the ethics committee approval cited above (AEŞH-BADEK-2025-0479); no separate data-access authorization was required. Secondary outcomes were high-risk mNUTRIC classification, individual and composite postoperative complications, and ICU and total hospital length of stay. The mentioned scoring systems and intensive care parameters were initially screened from the hospital patient database. When the database was found inadequate or missing data was observed, manually kept records were evaluated.

The mNUTRIC score was calculated using five variables: age, APACHE II score, SOFA score, number of comorbidities, and days from hospital admission to ICU admission. Because IL-6 levels were not routinely measured at our center, the original NUTRIC score could not be calculated, so the modified version validated by Rahman et al. [8] was used. Patients with a score of ≥5 were classified as having high nutritional risk. APACHE II scores were calculated within the first 24 h of ICU admission using the worst physiological values. SOFA scores were calculated daily, and the highest score during the first 48 h was recorded for analysis.

For the study sample, a power analysis was performed for the assessment of possible regression analysis by G*Power version 3.1.9.7 (Heinrich-Heine-Universität Düsseldorf, Düsseldort, Germany). To calculate the difference between two groups in a bivariate regression model, with a type 1 error of 0.05 (5%) and power of 80%, assuming equal allocation, at least 144 patients were required. For a multiple regression model, with the same type 1 error and power, and with an assumption of a possible 6 predictors in the model, at least 143 patients would be required. With the assumption of unequal distribution between groups and a possible data loss of 10%, at least 170 patients were planned to be enrolled in the study.

### Statistical Analysis

Patients’ data were first evaluated in Microsoft Office 365 Excel for any errors. Then, the data were transferred to IBM SPSS Statistics for Windows, version 31 (IBM Corp., Armonk, NY, USA) for further analysis. Descriptive statistics were reported as mean and standard deviation for normally distributed variables and as median with 25th to 75th percentiles for non-normal or asymmetric distributions. Categorical variables were compared using the Chi-square test, Phi, and Cramér’s V, based on group counts. When the distribution was unsuitable for Chi-square analysis, Fisher’s Exact Test was used. Scale parameters were compared using an independent samples *t*-test after assessing homogeneity of variance, and the resulting *p*-value was reported according to the homogeneity observed. Variables with nonparametric distributions were analyzed with the Mann–Whitney U test.

For parameters that were found to be statistically significant in either six-month all-cause mortality or nutritional risk, binomial regression models were developed to assess independent risk factors. Model performance was evaluated using the Hosmer–Lemeshow goodness-of-fit test and Nagelkerke R^2^. Variables with insufficient case numbers or high collinearity were to be excluded from the analysis. Receiver operating characteristic (ROC) analyses were conducted, with the Youden Index used to determine potential cutoff values for parameters identified as statistically significant in the regression analysis. Odds ratios (ORs) were reported with 95% confidence intervals. Statistical significance was set at *p* < 0.05.

ROC curves for admission serum albumin, the mNUTRIC score, and the NRS-2002 score were generated against six-month all-cause mortality; area-under-the-curve (AUC) values with 95% confidence intervals, sensitivities, specificities, positive predictive values, and negative predictive values at Youden-optimal cut-offs were reported for each marker, and AUCs were compared pairwise using DeLong’s test. As several variables used in the multivariable mortality model arise after the point of ICU admission and therefore may represent consequences of, rather than baseline predictors for, the outcome, a pre-specified sensitivity analysis was performed using a baseline-only model that retained variables measurable at the time of ICU admission (admission serum albumin, age, APACHE II on admission, admission vitamin D, and Charlson Comorbidity Index). ROC and regression analyses were performed in IBM SPSS Statistics version 31 (IBM Corp., Armonk, NY, USA); DeLong’s test for AUC comparison was performed in R version 4.5.3 (11 March 2026 ucrt) using the pROC package version 1.19.0.1 (R Foundation for Statistical Computing, Vienna, Austria).

## 3. Results

A total of 172 patients were included in the study. The mean age was 80.84 (±7.72) years, and the majority of patients were female (n = 116, 67.4%). The most common comorbidity was hypertension, present in 73.8% (n = 127) of patients, followed by coronary artery disease (CAD) (37.2%, n = 64) and diabetes mellitus (DM) (34.9%, n = 60). Proximal femur injury was the most common orthopedic pathology (77.9%, n = 134), and the distribution of operations was nearly equal across sides (Table 1).

Laboratory parameters were evaluated, and mean levels of hemoglobin, platelets, and white blood cells (WBC) were 9.9 g/L (±1.54), 219.49 × 10^9^/L (±80.47), and 11.7 × 10^9^/L (±4.02), respectively. Inflammatory parameters were elevated among patients, with a median procalcitonin (PRC) level of 0.21 ng/mL (0.12–0.38) and a mean C-reactive protein (CRP) level of 108.37 mg/L (±67.3). The mean creatinine and blood urea nitrogen (BUN) were 1.17 mg/L (±0.98) and 58.53 mg/L (±28.47), respectively, with no electrolyte imbalance observed. Venous blood gas values were within normal ranges, with a mean lactate level of 1.89 mmol/L (±0.58). Regarding the laboratory results, there were no differences between groups. Albumin levels were notably higher among survivors compared to non-survivors (33.49 ± 3.95 g/L vs. 30.31 ± 4.15 g/L, *p* = 0.001; Hedges’ g = 0.79, 95% CI 0.42 to 1.16), as were prealbumin levels (0.12 ± 0.05 g/L vs. 0.10 ± 0.05 g/L, *p* = 0.025; Hedges’ g = 0.41, 95% CI 0.05 to 0.77). Median vitamin D levels also tended to be higher in survivors (8 ng/mL vs. 6 ng/mL, *p* = 0.024; rank-biserial r = 0.24, 95% CI 0.09 to 0.38). Among the scoring systems, no statistically significant differences appeared in APACHE II (14.75 ± 5.49 vs. 16.38 ± 4.67, *p* = 0.094), NRS-2002 (1.41 ± 0.69 vs. 1.51 ± 0.64, *p* = 0.422), or mNUTRIC scores (4.28 ± 1.06 vs. 4.54 ± 1.02, *p* = 0.176) between survivors and non-survivors, while the median SOFA score was significantly higher among non-survivors (2 vs. 3, *p* = 0.001). Postoperative complications were more common among non-survivors, with significant differences seen for hypotension (3.8% vs. 30.8%), acute respiratory failure (7.5% vs. 38.5%), and sepsis (0.8% vs. 17.9%; all *p* = 0.001). Ward admission duration was similar between groups, but ICU length of stay (3.06 ± 1.57 vs. 5.19 ± 4.12 days, *p* = 0.004) and total hospital stay (8.41 ± 4.29 vs. 11.08 ± 5.7 days, *p* = 0.011) were significantly longer in non-survivors (Table 2).

Further analysis was performed by assessing patients according to mNUTRIC risk status, with 108 patients being classified in the low-risk group and 64 patients classified in the high-risk group. Patients in the high risk group were older (80.28 ± 8.21 years to 82.28 ± 6.65 years, *p* = 0.048), had a higher diagnosis rate of chronic renal disease (CRD) (n = 6, 5.6% to n = 12, 18.8%, *p* = 0.006) and congestive heart failure (CHF) (n = 7, 6.5% to n = 15, 23.4%, *p* = 0.001), lower mean hemoglobin (10.16 ± 1.58 g/dL to 9.48 ± 1.38 g/dL, *p* = 0.005) and median vitamin D (10 ng/mL to 7 ng/mL, *p* = 0.001) levels. Other comorbidities and laboratory results did not differ between groups (Table 3).

Patients in the high-risk group had a higher mean APACHE II score (12.52 ± 3.82 to 19.52 ± 4.65, *p* = 0.001) and a higher median SOFA score (2 to 3, *p* = 0.001). NRS scores did not vary between groups. Acute respiratory failure was the only postoperative complication that differed between groups and was more common among high-risk patients (n = 11, 10.2% to n = 14, 21.9%, *p* = 0.036). Intensive care unit admission was also longer among high-risk patients (3.06 ± 1.32 days to 4.36 ± 3.66 days, *p* = 0.010), and patients in the high-risk group had a higher rate of death during ICU admission (n = 4, 3.7% to n = 10, 15.6%, *p* = 0.006) (Table 4).

Parameters observed to differ between survivors and non-survivors were evaluated in a binomial regression model. In the model, albumin was included instead of prealbumin due to collinearity. Complications were evaluated as a group because the hypotension and acute respiratory failure groups had insufficient patients for a regression model. SOFA was also excluded due to collinearity and because its components were investigated in the same model. The omnibus likelihood-ratio test indicated that the model including the selected predictors explained significantly more variance in six-month all-cause mortality than a null (intercept-only) model (χ^2^ = 33.996, *p* < 0.001), with a Nagelkerke R^2^ of 0.292 and a non-significant Hosmer–Lemeshow goodness-of-fit test (*p* = 0.542), indicating adequate calibration. Overall, the model correctly classified 83.3% of the cases. Albumin and ICU admission duration were observed to be independent risk factors for mortality within six months (*p* values of 0.002 and 0.007, respectively). A similar model was created for possible factors affecting the nutritional risk group according to mNUTRIC. In this model, chronic renal disease and congestive heart failure were excluded due to inadequate patient count. Again, the omnibus likelihood-ratio test showed that this model explained significantly more variance in high-risk mNUTRIC classification than the null model (χ^2^ = 98.424, *p* < 0.001), with a Nagelkerke R^2^ of 0.619 and a non-significant Hosmer–Lemeshow test (*p* = 0.087), indicating acceptable model fit. The model correctly classified 82.2% of cases and identified age and APACHE II scores as independent risk factors for being in a high-risk nutritional status (Table 5).

**Figure 1:**
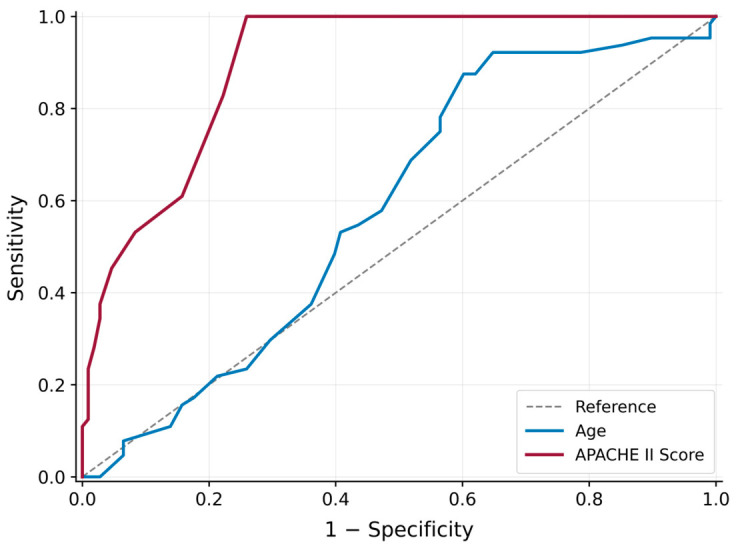
ROC Analysis of APACHE II and Age on Nutritional Risk Group Classification.

**Figure 2:**
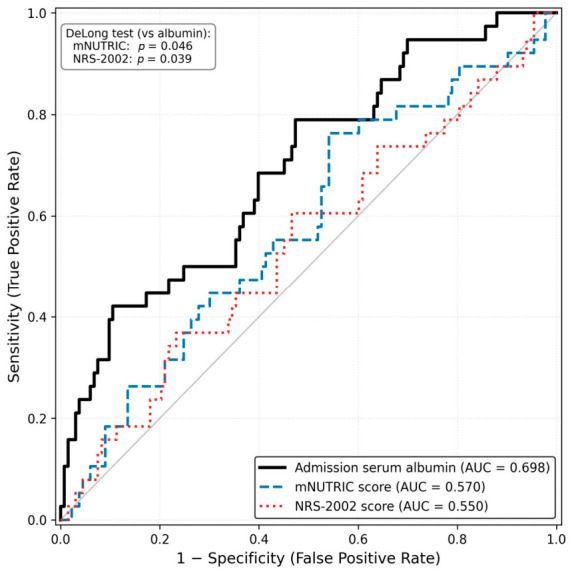
Combined Receiver Operating Characteristic (ROC) Curves for Admission Serum Albumin, the mNUTRIC Score, and the NRS-2002 Score for Six-Month All-Cause Mortality.

Because age and APACHE II are components of the mNUTRIC score itself, the preceding regression and ROC analyses for high-risk mNUTRIC classification are presented as a descriptive exploration of the score’s internal structure rather than as an independent prediction model. For completeness, the ROC analysis identified an APACHE II cut-off of 15 for high-risk mNUTRIC classification (AUC 0.896, 95% CI 0.851–0.941; sensitivity 82.81%, specificity 77.78%; age not statistically significant) (Figure 1). A head-to-head ROC analysis was performed to compare the discrimination of admission serum albumin, the mNUTRIC score, and the NRS-2002 score for six-month all-cause mortality in the 171 patients with complete data on all three markers (38 non-survivors). The AUC for admission serum albumin was 0.698 (95% CI 0.604–0.793), compared with 0.570 (95% CI 0.473–0.668) for mNUTRIC and 0.550 (95% CI 0.460–0.640) for NRS-2002. Pairwise comparison of AUCs using DeLong’s non-parametric test for correlated ROC curves showed a statistically significant difference between albumin and mNUTRIC (Z = 1.996, *p* = 0.046) and between albumin and NRS-2002 (Z = 2.069, *p* = 0.039), whereas the AUCs of mNUTRIC and NRS-2002 were not significantly different (Z = 0.335, *p* = 0.738). At the Youden-optimal cut-off of ≤29 g/L for admission serum albumin, sensitivity was 42.1% and specificity was 87.2% (positive predictive value 48.5%, negative predictive value 84.1%) for six-month all-cause mortality (Figure 2 and Table 6).

In the area under the curve (AUC) analysis, age was not statistically significant, whereas the APACHE II score was, with an AUC of 0.896 (0.851–0.941) and a *p*-value of 0.001.

A pre-specified sensitivity analysis was performed using a baseline-only multivariable model restricted to variables measurable at ICU admission. This model included admission serum albumin, age, APACHE II on admission, admission 25-hydroxy vitamin D, and the Charlson Comorbidity Index as predictors of six-month all-cause mortality (n = 166; 36 events). The model was statistically significant compared with the null model (χ^2^ = 23.482, df = 5, *p* < 0.001), with a Nagelkerke R^2^ of 0.203 and a non-significant Hosmer–Lemeshow test (*p* = 0.614), indicating acceptable calibration; the model correctly classified 81.3% of cases at a 0.5 probability cut-off. In this baseline-only model, admission serum albumin remained an independent predictor of six-month all-cause mortality (OR 0.830, 95% CI 0.747–0.923, *p* < 0.001), with an effect size essentially unchanged from that observed in the primary model (OR 0.823) (Table 7).

Age also emerged as an independent predictor when explicitly entered as a covariate (OR 1.062, 95% CI 1.005–1.122, *p* = 0.034). Admission APACHE II (OR 1.030, *p* = 0.473), admission 25-hydroxy vitamin D (OR 0.979, *p* = 0.432) and the Charlson Comorbidity Index (OR 0.973, *p* = 0.764) were not independent predictors (Table 7).

## 4. Discussion

The main finding of this study was that six-month all-cause mortality did not vary with comorbidity burden in older adults who had surgery for a simple fracture and later required ICU care. Similarly, fracture location and laterality had no impact on mortality. Although non-survivors were older, age was not retained in the primary multivariable mortality model because it is a component of the mNUTRIC score and was evaluated within the score rather than as an independent predictor; in the pre-specified baseline-only sensitivity analysis reported above, age was entered as an explicit covariate alongside admission serum albumin. Levels of albumin, prealbumin, and vitamin D were significantly lower in non-survivors, with albumin remaining statistically significant in the multivariate model. Other factors associated with six-month mortality included SOFA score, postoperative complications, and hospital stay length, with ICU length of stay being an independent risk factor. However, ICU and total hospital length of stay and postoperative complications accumulate after ICU admission and may therefore reflect consequences rather than independent baseline predictors of mortality. In the pre-specified sensitivity analysis, which restricted the model to admission-time variables, admission serum albumin remained independently associated with six-month all-cause mortality, with an essentially unchanged effect size (OR 0.830, 95% CI 0.747–0.923, *p* < 0.001). Age also emerged as an independent predictor (OR 1.062, 95% CI 1.005–1.122, *p* = 0.034) when entered as a covariate. Overall, admission serum albumin was the only one of the three candidate nutritional markers to show an independent association with six-month all-cause mortality in the multivariable model. The head-to-head ROC analysis showed significantly higher discrimination for albumin than for mNUTRIC (Z = 1.996, *p* = 0.046) or NRS-2002 (Z = 2.069, *p* = 0.039) on formal AUC comparison with DeLong’s test. The absolute AUC for albumin was nevertheless modest (0.698), and both composite nutritional scores performed close to chance (AUC 0.570 and 0.550, respectively), indicating that no single admission-time marker, including albumin, provides sufficient discrimination to be used as a stand-alone prognostic tool.

When patients were categorized by nutritional risk group, age remained a significant factor. CRD and CHF were more common in the high-risk group; however, due to limited patient numbers, these comorbidities could not be included in the regression analysis. Hemoglobin, albumin, and vitamin D levels were lower in the high-risk group, while creatinine levels were higher, likely reflecting the relationship between nutritional status and the burden of additional comorbidities. APACHE II and SOFA scores were higher in the high-risk group since these parameters are part of the mNUTRIC scoring system. Consistent with the mortality analysis, the high-risk group experienced more cases of acute respiratory failure and longer ICU stays. The regression analysis for nutritional risk group classification identified age and APACHE II as independent predictors, an expected finding given the significant role of these variables in the mNUTRIC scoring system. Notably, the study found an APACHE II cutoff of ≥15 as a potential value for detailed nutritional assessment in the ICU.

The study population showed a high prevalence of cardiovascular and metabolic comorbidities, typical of an orthogeriatric cohort. The predominance of proximal femur injuries aligns with the known epidemiology of frailty-related fractures in older adults. Laboratory results showed consistently elevated CRP and procalcitonin levels, likely indicating systemic inflammation and perioperative stress caused by trauma. Importantly, inflammatory markers did not differentiate survivors from non-survivors, suggesting that the differences observed in nutritional markers—including albumin and prealbumin—were not influenced by varying levels of inflammatory burden between groups.

The observation of albumin as an independent predictor of six-month mortality is consistent with the currently available literature. Bohl et al. stated that among geriatric hip fracture patients, preoperative serum albumin predicted survival and postoperative complications [16]. In a meta-analysis by Li et al., serum albumin levels were found to be associated with adverse outcomes after hip surgery, along with lymphocyte count and Mini Nutritional Assessment (MNA). It was stated that albumin remained the most frequently investigated and consistently significant across studies [17]. Gao et al., in a 2025 study, reported that an albumin cutoff of 33.9 g/L was associated with a threefold increase in one-year mortality among older adult patients with hip fractures [18]. The mean albumin values in our cohort (33.49 g/L in survivors and 30.31 g/L in non-survivors) are below the normal reference range and fall within the hypoalbuminemia range, indicating that patients in this study presented with some degree of reduced albumin at ICU admission. This is clinically important: while admission albumin was statistically associated with six-month mortality, the absolute between-group difference of approximately 3 g/L is modest, and the corresponding discrimination at the Youden-optimal cut-off of ≤29 g/L was characterized by a sensitivity of 42.1% and a positive predictive value of 48.5%. This would mean that almost six in ten decedents would be missed at this threshold. The relatively higher negative predictive value (84.1%) offers reasonable, but not absolute, reassurance when admission albumin is in the normal range. These operating characteristics underscore that admission albumin, although a useful and readily available prognostic signal in this orthogeriatric ICU population, should be interpreted as one component of a broader multidimensional assessment. Incorporating frailty, baseline functional status, comorbidity burden, and formal nutritional screening, thus, should be performed rather than albumin being utilized as a standalone tool. Albumin in this setting reflects a composite of two processes: pre-existing nutritional depletion in frail older adults and an acute-phase response to fracture and surgery. The consistently elevated CRP and procalcitonin values we observed, without a significant difference between survivors and non-survivors, argue that the inflammatory component is broadly similar across the cohort, which in turn suggests that the residual between-group difference in albumin partially reflects baseline nutritional reserve. Nevertheless, because albumin is not a specific nutritional marker, we would caution against interpreting our findings as a recommendation to use albumin alone as a nutritional assessment tool; rather, it should be considered a readily available prognostic marker whose interpretation must take inflammation into account [19].

Vitamin D levels were lower among non-survivors, although this was not observed in the multivariate model. These observations align with the study by Lee et al., which found vitamin D deficiency prevalent in the high-mortality group for hip surgery [20]. However, as in our study, no statistical significance was observed after regression analysis. Bayram et al. stated that vitamin D was independently associated with higher mortality among hip fracture patients, both as an independent marker and in a recent meta-analysis by Llombart et al. in 2024, which supported this observation yet noted possible heterogeneity [21,22]. These findings suggest that while vitamin D deficiency may serve as a marker of overall nutritional status, its independent contribution to mortality in the ICU setting remains debatable. Clinically, universal vitamin D supplementation protocols should be implemented as standard care in orthogeriatric ICU pathways.

It is also important to contextualize these findings with respect to clinical, rather than purely statistical, significance. The median 25-hydroxy vitamin D concentration in our cohort was 8 ng/mL overall (6 ng/mL in non-survivors, 8 ng/mL in survivors), which by every current consensus definition of vitamin D status places the entire cohort in the severely deficient range (<20 ng/mL and in most cases < 12 ng/mL). In this context, the statistically detectable difference between survivors and non-survivors of a few ng/mL is unlikely to translate into a clinically actionable discriminator, because irrespective of survival status, every patient in this population would warrant supplementation on the basis of their admission value alone. This so-called “ceiling” or “floor” effect, in which a biomarker shifts its entire distribution into an abnormal range, limits its utility for between-patient risk stratification even when group-level differences are statistically significant, and probably explains why vitamin D did not remain an independent predictor in the multivariate model. The broader clinical implication is that vitamin D screening and replacement should probably be regarded as a population-level intervention in orthogeriatric ICU patients rather than a tool for individual prognostication.

Our study found no statistically significant difference in mNUTRIC scores between survivors and non-survivors, although patients in the high-risk nutritional group had worse ICU outcomes. This observation was also reported by Xie et al. in 2025, in which an increase in mNUTRIC score was associated with higher overall mortality in the ICU [23]. This conflict in results may be related to differences in patient populations, asm NUTRIC was originally developed for critically ill patients rather than orthopedic patients. Wozniak et al. and de Vries et al. also support mNUTRIC in its role in assessing mortality in ICU settings [24,25]. Overall, we may state that while the mNUTRIC score is valuable in general ICU populations, its applicability to surgical orthogeriatric patients may require further calibration. NRS 2002 was not found to differ between survivors and non-survivors, unlike Schuetz et al.’s study, in which NRS 2002 scores were associated with increased mortality [26]. Supporting our results, an orthopedic-focused study by Inoue et al. found that NRS 2002 was outperformed by MNA and Geriatric Nutritional Risk Index, suggesting its possible limited sensitivity in the orthogeriatric setting [27].

SOFA was significantly higher among non-survivors, whereas APACHE II did not reach statistical significance. However, APACHE II was identified as an independent risk factor for being in the high-risk nutritional group at a cutoff of 15. This was similar to the study by Tian et al., which used a cutoff of 17 for the APACHE II score [28]. The combination of these scoring systems was also accepted as a valid approach, as reported by Falcao et al. [29]. However, it is important to emphasize that this finding does not mean that APACHE II functions as a nutritional screening tool in the classical sense. APACHE II was not developed to assess nutritional status, and it shares two components (age and the APACHE II score itself) with the mNUTRIC calculation. The association we observed between an APACHE II score ≥ 15 and a high-risk mNUTRIC classification is therefore best interpreted as an indirect observation that higher overall illness severity on ICU admission co-occurs with nutritional vulnerability in this orthogeriatric population, rather than as evidence that APACHE II independently captures nutritional status. We recommend APACHE II ≥ 15 serve as a prompt to initiate comprehensive nutritional assessment (e.g., NRS-2002, MNA-SF), rather than as a risk classifier itself. This threshold reflects illness severity co-occurring with nutritional vulnerability in orthogeriatric patients.

Based on the analytical assessment above, we do not recommend using an APACHE II score of ≥15 as a stand-alone clinical trigger for nutritional risk assessment in orthogeriatric ICU patients. Our findings, as an external and methodologically distinct observation, support the co-occurrence of higher overall illness severity on ICU admission and nutritional vulnerability in this population, consistent with routine clinical practice in which high-illness-severity patients already receive early nutrition-specialist involvement. Rather than proposing a specific APACHE II cut-off, we would suggest that validated nutritional screening (for example, NRS-2002 at admission, with a more detailed geriatric nutritional assessment where indicated) be applied to every older adult admitted to the ICU after fracture surgery. Prospective multicenter studies with frailty, functional, and nutritional covariates are needed to determine whether any single admission-time biomarker provides additional discrimination beyond structured nutritional assessment.

The association between prolonged ICU admission and six-month mortality observed in our study is well-supported in the literature. Bani Essa et al. stated that prolonged hospitalization among older adult hip fracture patients was an independent risk factor for one-year mortality [30]. A study by Arslan et al. in a Turkish cohort reported that the prognostic nutritional index at admission was predictive of both ICU requirement and mortality in geriatric hip fracture patients, linking nutritional status directly to the need for and duration of intensive care [31]. These results support the clinical importance of minimizing ICU admission duration through early nutritional optimization and perioperative care pathways.

Recent large-scale studies have continued to affirm the detrimental impact of malnutrition on outcomes in older adult trauma patients. Coaston et al. found that malnutrition in older trauma patients was associated with increased mortality and clinical complications [32]. A systematic review and meta-analysis by Chiavarini et al. reported that malnutrition increased the risk of adverse outcomes during long term follow-up, with up to 250% at one year among older adult hip fracture patients [33]. These findings are consistent with our study’s overall conclusion that nutritional status, whether assessed by individual biomarkers such as albumin or by scoring systems, plays a central role in determining outcomes after fracture surgery in the older adults.

The study’s main limitation was its retrospective design and single-center setting, which may introduce selection bias and limit how broadly our findings can be applied. A prospective study could have provided additional data for overall survival analysis. A multicenter study would have included more patients, potentially capturing a wider range of complications and comorbidities. Additionally, although our overall sample size satisfied the requirements of the power analysis, it was not large enough to conduct robust subgroup analyses on relatively rare outcomes. As a result, specific comorbidities (such as chronic renal disease and congestive heart failure) and individual postoperative complications (such as hypotension and sepsis) had to be excluded from the regression models due to insufficient patient numbers, which may have concealed potential associations. Several additional limitations warrant specific emphasis. First, our primary multivariable mortality model included ICU length of stay, total hospital length of stay, and composite postoperative complications as covariates. These variables are not available at the point of ICU admission and, as they develop over the course of the clinical course, may represent consequences of, rather than independent baseline predictors for, mortality. We addressed this by pre-specifying a baseline-only sensitivity analysis restricted to admission-time variables (reported above); nonetheless, the main mortality model should be interpreted with this limitation in mind and is better viewed as an associative, rather than an early-prediction, tool. Second, our analysis of predictors of high-risk mNUTRIC classification is subject to analytical circularity because age, APACHE II and SOFA are themselves components of the mNUTRIC score. The apparent independent association of these variables with high-risk mNUTRIC classification, and the corresponding APACHE II ≥ 15 threshold, therefore reflect the internal structure of the score rather than a genuinely novel prognostic relationship. Third, several clinically important variables known to influence outcomes in orthogeriatric ICU patients were not routinely captured in the hospital dataset and could not be included in our analysis. In addition, the events-per-variable ratio in our primary multivariable mortality model was approximately 6.6 which is below the conventional threshold of 10 events per variable. Although the pre-specified baseline-only sensitivity analysis yielded an essentially unchanged effect size for admission serum albumin (OR 0.830 vs. primary OR 0.823), regression estimates from the primary model should be interpreted with this caveat, and validation in a larger cohort is warranted. Fourth, the modest absolute albumin difference and the Youden-optimal cut-off of ≤29 g/L (sensitivity 42.1%, specificity 87.2%) suggest limited standalone screening utility. Future studies should evaluate whether combining albumin with frailty indices improves discrimination beyond AUC 0.698. These include validated frailty indices, admission functional status, anthropometric measures, dietary intake prior to admission and geriatric-specific complications such as delirium and in-hospital falls.

## 5. Conclusions

In this single-center retrospective cohort of older adult ICU patients admitted after simple fracture surgery, admission serum albumin was the only one of three candidate nutritional markers (admission serum albumin, the mNUTRIC score, and NRS-2002) that showed an independent association with six-month all-cause mortality in multivariable analysis; neither the mNUTRIC score nor NRS-2002 discriminated between survivors and non-survivors, and the formal head-to-head comparison of AUCs using DeLong’s test is reported in the Results. These results should be interpreted cautiously: the between-group albumin difference was modest in absolute terms, all patients were vitamin D-deficient (limiting vitamin D as an individual-level discriminator), and the primary multivariable model included variables accrued after ICU admission that may represent consequences of severity rather than baseline predictors. The apparent association between APACHE II and high-risk mNUTRIC classification reflects the internal structure of the mNUTRIC score rather than an independent clinical finding and is not proposed as a nutritional screening recommendation. Prospective multi-center studies that prospectively capture frailty and nutritional covariates are needed to determine whether admission serum albumin adds incremental prognostic value beyond validated nutritional screening in orthogeriatric ICU patients.

## Figures and Tables

**Table 1:** Demographic Characteristics, Comorbidities, and Orthopedic Assessment According to Six-Month All-Cause Mortality.

Parameters (n, %)		Survivors (n = 133)	Non-Survivors (n = 39)	Total (n = 172)	*p* Value
Sex	Male	42 (31.6)	14 (35.9)	56 (32.6)	0.613
	Female	91 (68.4)	25 (64.1)	116 (67.4)	
Age (years, SD)		80.12 (7.72)	83.33 (7.29)	80.84 (7.72)	0.022
**Comorbidity Assessment**					
Charlson Comorbidity Index Score (SD)		5.01 (2.34)	5.26 (2.18)	5.07 (2.30)	0.56
Hypertension		100 (75.2)	27 (69.2)	127 (73.8)	0.457
Diabetes Mellitus		43 (32.3)	17 (43.6)	60 (34.9)	0.195
Cerebrovascular Event		14 (10.5)	6 (15.4)	20 (11.6)	0.283 *
Alzheimer’s Disease		20 (15)	7 (17.9)	27 (15.7)	0.66
Chronic Renal Disease		15 (11.3)	3 (7.7)	18 (10.5)	0.380 *
Congestive Heart Failure		17 (12.8)	5 (12.8)	22 (12.8)	0.591 *
Malignancy		9 (6.8)	5 (12.8)	14 (8.1)	0.185 *
Coronary Artery Disease		50 (37.6)	14 (35.9)	64 (37.2)	0.847
Atrial Fibrillation		10 (7.5)	5 (12.8)	15 (8.7)	0.232 *
COPD		22 (16.5)	2 (5.1)	24 (14)	0.07
Parkinson’s Disease		6 (4.5)	0 (0)	6 (3.5)	0.308 *
Hypothyroidism		16 (12)	4 (10.3)	20 (11.6)	0.509 *
Rheumatoid Arthritis		1 (0.8)	0 (0)	1 (0.6)	0.773 *
Cirrhosis		1 (0.8)	1 (2.6)	2 (1.2)	0.403 *
**Orthopedic Assessment**					
Fracture Localization	Proximal Femur	103 (77.4)	31 (79.5)	134 (77.9)	0.396
	Femur Shaft	5 (3.8)	4 (10.3)	9 (5.2)	
	Distal Femur	1 (0.8)	0 (0)	1 (0.6)	
	Humerus	10 (7.5)	1 (2.6)	11 (6.4)	
	Distal Humerus	2 (1.5)	0 (0)	2 (1.2)	
	Forearm	2 (1.5)	0 (0)	2 (1.2)	
	Proximal Tibia	1 (0.8)	1 (2.6)	2 (1.2)	
	Tibia Shaft	2 (1.5)	0 (0)	2 (1.2)	
	Acetabulum	1 (0.8)	0 (0)	1 (0.6)	
	Ankle	4 (3)	0 (0)	4 (2.3)	
	Patella	1 (0.8)	0 (0)	1 (0.6)	
	Periprosthetic	1 (0.8)	2 (5.1)	3 (1.7)	
Fracture Side	Left	67 (51.9)	18 (47.4)	85 (50.9)	0.62
	Right	62 (48.1)	20 (52.6)	82 (49.1)	

SD: Standard Deviation, COPD: Chronic Obstructive Pulmonary Disease. Groups were compared using the Chi-Square test and the independent-samples *t*-test. * Groups were compared with Fisher’s Exact Test. Six-month all-cause mortality refers to death from any cause within 180 days of ICU admission.

**Table 2:** Laboratory Values, Scoring Systems, Complications, and Admission Evaluation.

Parameters (n, SD)		Survivors (n = 133)	Non-Survivors (n = 39)	Total (n = 172)	*p* Value
Hemoglobin (g/dL)		9.99 (1.61)	9.62 (1.26)	9.9 (1.54)	0.194
Platelets (×10^9^/L)		216.7 (77.77)	229.24 (89.72)	219.49 (80.47)	0.399
White Blood Cells (×10^9^/L)		11.64 (4.11)	11.92 (3.7)	11.7 (4.02)	0.705
PRC (ng/mL) (median, 25th–75th)		0.20 (0.1–0.37)	0.26 (0.16–0.47)	0.21 (0.12–0.38)	0.051 **
C-Reactive Protein (mg/L)		108.29 (71.56)	108.66 (50.4)	108.37 (67.3)	0.976 *
Creatinine (mg/L)		1.2 (1.04)	1.05 (0.7)	1.17 (0.98)	0.387
Blood Urea Nitrogen (mg/L)		58.44 (29.99)	58.83 (22.89)	58.53 (28.47)	0.941
Sodium (mEq/L)		138.64 (3.26)	138.72 (4.46)	138.66 (3.55)	0.919 *
Potassium (mEq/L)		4.27 (0.47)	4.15 (0.52)	4.24 (0.48)	0.166
Calcium (mg/L)		8.23 (0.54)	8.15 (0.52)	8.21 (0.53)	0.411
Magnesium (mg/L)		1.91 (0.28)	1.89 (0.21)	1.91 (0.26)	0.6
Albumin (g/L)		33.49 (3.95)	30.31 (4.15)	32.78 (4.2)	0.001
pH		7.39 (0.05)	7.39 (0.08)	7.39 (0.06)	0.693 *
Pco2 (mmHg)		35.43 (6.51)	35.53 (8.3)	35.45 (6.93)	0.938
HCO3 (mEq/L)		21.21 (3.01)	21.82 (4.15)	21.35 (3.3)	0.315
Lactate (mmol/L)		1.89 (0.61)	1.92 (0.44)	1.89 (0.58)	0.721 *
Prealbumin (g/L)		0.12 (0.05)	0.1 (0.05)	0.11 (0.05)	0.025
Vitamin D (ng/mL) (median, 25th–75th)		8 (5–18)	6 (3–12)	8 (4–17)	0.024 **
**ICU and Nutritional Scoring**					
APACHE II (mean, SD)		14.75 (5.49)	16.38 (4.67)	15.12 (5.35)	0.094
SOFA (median, 25th–75th)		2 (1–3)	3 (2–4)	2 (1–3)	0.001 **
NRS-2002 (mean, SD)		1.41 (0.69)	1.51 (0.64)	1.44 (0.68)	0.422
mNUTRIC (mean, SD)		4.28 (1.06)	4.54 (1.02)	4.34 (1.06)	0.176
mNUTRIC Classification (n, %)	Low Risk	87 (65.4)	21 (53.8)	108 (62.8)	0.189
	High Risk	46 (34.6)	18 (46.2)	64 (37.2)	
**Post Operative Complications** (n, %)					
Any Complication		56 (42.1)	26 (66.7)	82 (47.7)	0.007
Acute Renal Failure		21 (15.8)	10 (25.6)	31 (18)	0.159
Hemorrhage		30 (22.6)	9 (23.1)	39 (22.7)	0.946
Hypotension		5 (3.8)	12 (30.8)	17 (9.9)	0.001
Acute Respiratory Failure		10 (7.5)	15 (38.5)	25 (14.5)	0.001
Sepsis		1 (0.8)	7 (17.9)	8 (4.7)	0.001 ***
**Admission Evaluation** (Days, SD)					
ICU Admission		3.06 (1.57)	5.19 (4.12)	3.53 (2.52)	0.004 *
Ward Admission		5.09 (3.38)	5.67 (4.04)	5.22 (3.54)	0.371
Total Hospital Admission		8.41 (4.29)	11.08 (5.7)	9 (4.76)	0.011 *
Ventilation Support Duration (Days)		5 (0)	4.67 (3.08)	4.69 (2.95)	0.919

SD: Standard Deviation, PRC: Procalcitonin, ICU: Intensive Care Unit, SOFA: Sequential Organ Failure Assessment, NRS-2002: Nutrition Risk Screening 2002, mNUTRIC: Modified Nutrition Risk in the Critically Ill. * Homogeneity of variance was not equal. ** Independent-Samples Mann–Whitney U Test was performed for groups with non-parametric distribution. *** Fisher’s Exact *t*-test was utilized for comparison. Six-month all-cause mortality refers to death from any cause within 180 days of ICU admission.

**Table 3:** Comorbidities and Laboratory Values According to Nutritional Status.

Parameters (n, %)		Low Risk (n = 108)	High Risk (n = 64)	Total (n = 172)	*p* Value
Sex	Male	33 (30.6)	23 (35.9)	56 (32.6)	0.467
	Female	75 (69.4)	41 (64.1)	116 (67.4)	
Age (years, SD)		80 (8.21)	82.28 (6.65)	80.85 (7.72)	0.048 **
**Comorbidity Assessment**					
Charlson Comorbidity Index Score		4.89 (2.38)	5.38 (2.16)	5.07 (2.31)	
Hypertension		79 (73.1)	48 (75)	127 (73.8)	0.789
Diabetes Mellitus		32 (29.6)	28 (43.8)	60 (34.9)	0.06
Cerebrovascular Event		13 (12)	7 (10.9)	20 (11.6)	0.828
Alzheimer’s Disease		17 (15.7)	10 (15.6)	27 (15.7)	0.984
Chronic Renal Disease		6 (5.6)	12 (18.8)	18 (10.5)	0.006
Congestive Heart Failure		7 (6.5)	15 (23.4)	22 (12.8)	0.001
Malignancy		8 (7.4)	6 (9.4)	14 (8.1)	0.648
Coronary Artery Disease		39 (36.1)	25 (39.1)	64 (37.2)	0.699
Atrial Fibrillation		7 (6.5)	8 (12.5)	15 (8.7)	0.176
COPD		19 (17.6)	5 (7.8)	24 (14)	0.074
Parkinson’s Disease		5 (4.6)	1 (1.6)	6 (3.5)	0.414 *
Hypothyroidism		13 (12)	7 (10.9)	20 (11.6)	0.828
Rheumatoid Arthritis		1 (0.9)	0 (0)	1 (0.6)	0.628 *
Cirrhosis		1 (0.9)	1 (1.6)	2 (1.2)	0.607 *
**Laboratory Parameters** (mean, SD)					
Hemoglobin (g/dL)		10.16 (1.58)	9.48 (1.38)	9.9 (1.54)	0.005
Platelets (×10^9^/L)		219.26 (79.18)	219.87 (83.28)	219.49 (80.47)	0.962
White Blood Cells (×10^9^/L)		11.8 (3.61)	11.53 (4.64)	11.7 (4.02)	0.673
PRC (ng/mL)		0.20 (0.1–0.33)	0.25 (0.13–0.43)	0.21 (0.12–0.38)	0.157 ***
C-Reactive Protein (mg/L)		114.43 (70.96)	97.97 (59.64)	108.37 (67.3)	0.123
Creatinine (mg/L)		1 (0.49)	1.45 (1.43)	1.17 (0.98)	0.017 **
Blood Urea Nitrogen (mg/L)		54.61 (25.42)	65.24 (32.17)	58.53 (28.47)	0.027 **
Sodium (mEq/L)		138.7 (3.21)	138.58 (4.08)	138.66 (3.55)	0.823
Potassium (mEq/L)		4.19 (0.46)	4.32 (0.52)	4.24 (0.48)	0.098
Calcium (mg/L)		8.25 (0.53)	8.14 (0.54)	8.21 (0.53)	0.174
Magnesium (mg/L)		1.9 (0.25)	1.92 (0.29)	1.91 (0.26)	0.565
Albumin (g/L)		33.34 (4.11)	31.83 (4.21)	32.78 (4.2)	0.023
Prealbumin (g/L)		0.12 (0.05)	0.11 (0.06)	0.11 (0.05)	0.138
Vitamin D (ng/mL)		10 (5–18)	7 (3–11)	8 (4–17)	0.001 ***

SD: Standard Deviation, PRC: Procalcitonin, COPD: Chronic Obstructive Pulmonary Disease. * Fisher’s Exact *t*-test was utilized for comparison. ** Homogeneity of variance was not equal. *** Independent-Samples Mann–Whitney U Test was performed for groups with non-parametric distribution. PRC and Vitamin D values were given with median and 25th to 75th percentiles.

**Table 4:** Scoring Systems, Complications, and Admission Evaluation According to Nutritional Status.

Parameters (Mean, SD)		Low Risk (n = 108)	High Risk (n = 64)	Total (n = 172)	*p* Value
APACHE II		12.52 (3.82)	19.52 (4.65)	15.12 (5.35)	0.001 *
SOFA (median, 25th–75th)		2 (1–3)	3 (2–4)	2 (1–3)	0.001 **
NRS 2002		1.38 (0.64)	1.53 (0.73)	1.44 (0.68)	0.172 *
**Post Operative Complications** (n, %)					
Any Complication		48 (44.4)	34 (53.1)	82 (47.7)	0.271
Acute Renal Failure		17 (15.7)	14 (21.9)	31 (18)	0.312
Hemorrhage		25 (23.1)	14 (21.9)	39 (22.7)	0.847
Hypotension		7 (6.5)	10 (15.6)	17 (9.9)	0.052
Acute Respiratory Failure		11 (10.2)	14 (21.9)	25 (14.5)	0.036
Sepsis		2 (1.9)	6 (9.4)	8 (4.7)	0.051 ***
**Admission Evaluation** (Days, SD)					
Intensive Care Unit Admission Duration		3.06 (1.32)	4.34 (3.66)	3.53 (2.52)	0.010 *
Ward Admission Duration		5.07 (3.48)	5.48 (3.66)	5.22 (3.54)	0.47
Total Hospital Admission Duration		8.55 (4.6)	9.79 (4.96)	9 (4.76)	0.105
Ventilation Support Requirement Duration (Days)		3 (2)	5.2 (3.08)	4.69 (2.95)	0.276
Intensive Care Unit Discharge	Non-Survivor	4 (3.7)	10 (15.6)	14 (8.1)	0.006
	Discharged	104 (96.3)	54 (84.4)	158 (91.9)	

SD: Standard Deviation, SOFA: Sequential Organ Failure, NRS 2002: Nutrition Risk Screening 2002. * Homogeneity of variance was not equal. ** Independent-Samples Mann–Whitney U Test was performed for groups with non-parametric distribution. *** Fisher’s Exact *t*-test was utilized for comparison.

**Table 5:** Binomial Regression Analyses for Mortality within Six Months and mNUTRIC Risk Assessment.

Mortality Within Six Months	B	S.E.	Wald	*p* Value	Odds Ratio
Albumin	−0.195	0.062	10.025	0.002	0.823 (0.729–0.928)
Vitamin D	−0.040	0.029	1.966	0.161	0.961 (0.909–1.016)
Post Operative Complication	−0.062	0.482	0.017	0.897	0.940 (0.366–2.415)
ICU Admission Duration	0.345	0.127	7.396	0.007	1.413 (1.101–1.812)
Total Hospital Admission Duration	−0.018	0.051	0.130	0.718	0.982 (0.889–1.085)
Constant	4.323	2.242	3.718	0.054	75.440
**mNUTRIC Risk Group**					
Age	0.111	0.034	10.466	0.001	1.117 (1.045–1.195)
Hemoglobin	0.034	0.169	0.040	0.842	1.034 (0.743–1.439)
Vitamin D	0.018	0.029	0.393	0.531	1.018 (0.962–1.077)
APACHE II	0.527	0.091	33.384	0.001	1.694 (1.417–2.026)
SOFA	0.019	0.161	0.015	0.904	1.02 (0.743–1.399)
Acute Respiratory Failure	0.633	0.819	0.596	0.440	1.883 (0.378–9.383)
ICU Admission Duration	0.139	0.123	1.293	0.255	1.15 (0.904–1.462)
Total Hospital Admission Duration	0.022	0.052	0.184	0.668	1.023 (0.923–1.133)
Constant	−19.783	4.614	18.379	0.001	0.001

S.E.: Standard Error, mNUTRIC: Modified Nutrition Risk in the Critically Ill, ICU: Intensive Care Unit, SOFA: Sequential Organ Failure. Odds ratios were given with 95% confidence interval for lower and upper bound values. Six-month all-cause mortality refers to death from any cause within 180 days of ICU admission.

**Table 6:** Admission Serum Albumin, mNUTRIC, and NRS-2002 for Six-Month All-Cause Mortality.

Marker	AUC (95% CI)	Youden Cut	Sens (%)	Spec (%)	PPV (%)	NPV (%)	DeLong vs. Albumin (*p* Value)
Admission serum albumin	0.698 (0.604–0.793)	≤29.0	42.1	87.2	48.5	84.1	—
mNUTRIC score	0.570 (0.473–0.668)	>5	44.7	65.4	27	80.6	0.046
NRS-2002 score	0.550 (0.460–0.640)	>2	44.7	64.7	26.6	80.4	0.039

AUC: Area Under the Curve; CI: Confidence Interval; Sens: Sensitivity; Spec: Specificity; PPV: Positive Predictive Value; NPV: Negative Predictive Value. Six-month all-cause mortality refers to death from any cause within 180 days of ICU admission.

**Table 7:** Baseline-Only Multivariable Logistic Regression for Six-Month All-Cause Mortality.

Variable	B	SE	Wald	*p*	OR (95% CI)
Admission serum albumin (g/L)	−0.186	0.054	11.953	0.001	0.830 (0.747–0.923)
Age (years)	0.06	0.028	4.514	0.034	1.062 (1.005–1.122)
Admission APACHE II	0.03	0.041	0.516	0.473	1.030 (0.950–1.116)
Admission 25-OH vitamin D (ng/mL)	−0.021	0.027	0.617	0.432	0.979 (0.929–1.032)
Charlson Comorbidity Index	−0.027	0.09	0.09	0.764	0.973 (0.816–1.161)
Constant	−0.370	3.06	0.015	0.904	0.691

B: regression coefficient; SE: standard error; Wald: Wald χ^2^ statistic; OR: Odds Ratio; CI: Confidence Interval. Six-month all-cause mortality refers to death from any cause within 180 days of ICU admission.

## Data Availability

The data presented in this study are available on request from the corresponding author, after approval from the ethics committee as per data sharing and personal data security laws of Türkiye.

## Abbreviations

The following abbreviations are used in this manuscript:

APACHE II	Acute Physiology and Chronic Health Evaluation II
AUC	Area Under the Curve
BMI	Body Mass Index
BUN	Blood Urea Nitrogen
CAD	Coronary Artery Disease
CHF	Congestive Heart Failure
CI	Confidence Interval
CRD	Chronic Renal Disease
CRP	C-Reactive Protein
DM	Diabetes Mellitus
ICU	Intensive Care Unit
IL-6	Interleukin-6
MNA	Mini Nutritional Assessment
mNUTRIC	Modified Nutrition Risk in the Critically Ill
MUST	Malnutrition Universal Screening Tool
NRS-2002	Nutrition Risk Screening 2002
OR	Odds Ratio
PRC	Procalcitonin
ROC	Receiver Operating Characteristic
SOFA	Sequential Organ Failure Assessment
WBC	White Blood Cells

## References

[1] Hegazi R., Miller A., Sauer A. (2024). Evolution of the Diagnosis of Malnutrition in Adults: A Primer for Clinicians. Front. Nutr..

[2] Williams D.G.A., Molinger J., Wischmeyer P.E. (2019). The Malnourished Surgery Patient. Curr. Opin. Anaesthesiol..

[3] Uhl S., Siddique S.M., Bloschichak A., McKeever W., D’Anci K., Leas B., Mull N.K., Compher C., Lewis J.D., Wu G.D. (2022). Interventions for Malnutrition in Hospitalized Adults: A Systematic Review and Meta-Analysis. J. Hosp. Med..

[4] McClave S.A., DiBaise J.K., Mullin G.E., Martindale R.G. (2016). ACG Clinical Guideline: Nutrition Therapy in the Adult Hospitalized Patient. Am. J. Gastroenterol..

[5] Cederholm T., Barazzoni R., Austin P., Ballmer P., Biolo G., Bischoff S.C., Compher C., Correia I., Higashiguchi T., Holst M. (2017). ESPEN Guidelines on Definitions and Terminology of Clinical Nutrition. Clin. Nutr..

[6] Kondrup J. (2003). Nutritional Risk Screening (NRS 2002): A New Method Based on an Analysis of Controlled Clinical Trials. Clin. Nutr..

[7] Heyland D.K., Dhaliwal R., Jiang X., Day A.G. (2011). Identifying Critically Ill Patients Who Benefit the Most from Nutrition Therapy: The Development and Initial Validation of a Novel Risk Assessment Tool. Crit. Care.

[8] Rahman A., Hasan R.M., Agarwala R., Martin C., Day A.G., Heyland D.K. (2016). Identifying Critically-Ill Patients Who Will Benefit Most from Nutritional Therapy: Further Validation of the “Modified NUTRIC” Nutritional Risk Assessment Tool. Clin. Nutr..

[9] Liu H.-T., Wu S.-C., Tsai C.-H., Li C., Chou S.-E., Su W.-T., Hsu S.-Y., Hsieh C.-H. (2020). Association Between Geriatric Nutritional Risk Index and Mortality in Older Trauma Patients in the Intensive Care Unit. Nutrients.

[10] Cross M.B., Yi P.H., Thomas C.F., Garcia J., Della Valle C.J. (2014). Evaluation of Malnutrition in Orthopaedic Surgery. J. Am. Acad. Orthop. Surg..

[11] Cacciola G., Mancino F., Holzer L.A., De Meo F., De Martino I., Bruschetta A., Risitano S., Sabatini L., Cavaliere P. (2023). Predictive Value of the C-Reactive Protein to Albumin Ratio in 30-Day Mortality After Hip Fracture in Elderly Population: A Retrospective Observational Cohort Study. J. Clin. Med..

[12] Meermans G., van Egmond J.C. (2025). Malnutrition in Older Hip Fracture Patients: Prevalence, Pathophysiology, Clinical Outcomes, and Treatment—A Systematic Review. J. Clin. Med..

[13] Akcaalan S., Akbulut B., Memis K., Caglar C., Ugurlu M., Kapicioglu M.I.S., Dogan M. (2025). The Akcaalan Mortality Score: A Novel Mortality Score to Predict 3-Year Mortality for Elderly Hip Fractures. J. Clin. Med..

[14] Portuondo J.I., Probstfeld L., Massarweh N.N., Le L., Wei Q., Chai C.Y., Taylor J., Awad S.S., Tran Cao H.S. (2020). Malnutrition in Elective Surgery: How Traditional Markers Might Be Failing Surgeons and Patients. Surgery.

[15] Di Filippo L., Terenzi U., Giustina A. (2025). Vitamin D in the elderly: The phil-rouge in preventing bone, muscle and adipose deterioration?. Arch. Endocrinol. Metab..

[16] Bohl D.D., Shen M.R., Hannon C.P., Fillingham Y.A., Darrith B., Della Valle C.J. (2017). Serum Albumin Predicts Survival and Postoperative Course Following Surgery for Geriatric Hip Fracture. J. Bone Jt. Surg..

[17] Li S., Zhang J., Zheng H., Wang X., Liu Z., Sun T. (2019). Prognostic Role of Serum Albumin, Total Lymphocyte Count, and Mini Nutritional Assessment on Outcomes After Geriatric Hip Fracture Surgery: A Meta-Analysis and Systematic Review. J. Arthroplast..

[18] Gao Y., Zhou S., Gao W., Zhang Y., Shi L., Xie T., Tian C., Chen H., Rui Y. (2025). Preoperative Indicators for 1-Year Mortality in Elderly Individuals Following Hip Fracture Surgery Under A Multidisciplinary Team Co-Management Model: A Single-Centre Retrospective Observational Study. Geriatr. Orthop. Surg. Rehabil..

[19] Yao W., Tang W., Wang W., Lv Q., Ding W. (2023). Correlation Between Admission Hypoalbuminemia and Postoperative Urinary Tract Infections in Elderly Hip Fracture Patients. J. Orthop. Surg. Res..

[20] Lee G.-H., Lim J.-W., Park Y.-G., Ha Y.-C. (2015). Vitamin D Deficiency Is Highly Concomitant but Not Strong Risk Factor for Mortality in Patients Aged 50 Year and Older with Hip Fracture. J. Bone Metab..

[21] Llombart R., Mariscal G., Barrios C., de la Rubia Ortí J.E., Llombart-Ais R. (2024). Impact of Vitamin D Deficiency on Mortality in Patients with Hip Fracture: A Meta-analysis. J. Am. Geriatr. Soc..

[22] Bayram J.M., Kanesan H., Clement N.D. (2024). Vitamin D Deficiency in Hip Fracture Patients Is Associated with an Increased Mortality Risk. Eur. J. Orthop. Surg. Traumatol..

[23] Xie M., Huang L., Li L., Qin Y., Feng B., Cai Q., Huang D. (2025). Association between NUTRIC Score and ICU Mortality in Patients with Sepsis: A Prospective Cohort Study. Front. Nutr..

[24] de Vries M.C., Koekkoek W., Opdam M.H., van Blokland D., van Zanten A.R. (2018). Nutritional Assessment of Critically Ill Patients: Validation of the Modified NUTRIC Score. Eur. J. Clin. Nutr..

[25] Wełna M., Adamik B., Kübler A., Goździk W. (2023). The NUTRIC Score as a Tool to Predict Mortality and Increased Resource Utilization in Intensive Care Patients with Sepsis. Nutrients.

[26] Schuetz P., Sulo S., Walzer S., Vollmer L., Stanga Z., Gomes F., Rueda R., Mueller B., Partridge J. (2020). Economic Evaluation of Individualized Nutritional Support in Medical Inpatients: Secondary Analysis of the EFFORT Trial. Clin. Nutr..

[27] Inoue T., Misu S., Tanaka T., Kakehi T., Ono R. (2019). Acute Phase Nutritional Screening Tool Associated with Functional Outcomes of Hip Fracture Patients: A Longitudinal Study to Compare MNA-SF, MUST, NRS-2002 and GNRI. Clin. Nutr..

[28] Tian Y., Yao Y., Zhou J., Diao X., Chen H., Cai K., Ma X., Wang S. (2022). Dynamic APACHE II Score to Predict the Outcome of Intensive Care Unit Patients. Front. Med..

[29] Falcão A.L.E., de Almeida Barros A.G., Bezerra A.A.M., Ferreira N.L., Logato C.M., Silva F.P., do Monte A.B.F.O., Tonella R.M., de Figueiredo L.C., Moreno R. (2019). The Prognostic Accuracy Evaluation of SAPS 3, SOFA and APACHE II Scores for Mortality Prediction in the Surgical ICU: An External Validation Study and Decision-Making Analysis. Ann. Intensive Care.

[30] Essa S., Anaqreh Y., Abueed M., Alrawashdeh M., Hussein N., Al-Sa’adi Y., Batbouta J., Alkhatatba M., Mohaidat Z., Radaideh A. (2024). Impact of Surgical Timing on Mortality and Functional Outcomes in Elderly Hip Fracture Patients: A Retrospective Cohort Study. Acta Inform. Medica.

[31] Arslan K. (2024). Predictive Value of Prognostic Nutritional Index on Postoperative Intensive Care Requirement and Mortality in Geriatric Hip Fracture Patients. North Clin. Istanb..

[32] Coaston T.N., Ali K., Vadlakonda A., Mehta D.J., Sakowitz S., Yalzadeh D., Tillou A., Benharash P. (2025). The Association of Malnutrition with Clinical and Financial Outcomes of Traumatic Injuries in Older Adults: A National Retrospective Analysis. Surg. Open Sci..

[33] Chiavarini M., Ricciotti G.M., Genga A., Faggi M.I., Rinaldi A., Toscano O.D., D’Errico M.M., Barbadoro P. (2024). Malnutrition-Related Health Outcomes in Older Adults with Hip Fractures: A Systematic Review and Meta-Analysis. Nutrients.

